# A new species of *Enterognathus* (Copepoda, Cyclopoida, Enterognathidae) collected from the Seto Inland Sea, western Japan

**DOI:** 10.3897/zookeys.180.2509

**Published:** 2012-04-05

**Authors:** Susumu Ohtsuka, Michitaka Shimomura, Kota Kitazawa

**Affiliations:** 1 Takehara Marine Science Station, Setouchi Field Science Center, Hiroshima University, 5-8-1 Minato-machi, Takehara, Hiroshima 725-0024, Japan; 2 Kitakyushu Museum of Natural History and Human History, Kitakyushu 805-0071, Japan; 3 Atmosphere and Ocean Research Institute, the University of Tokyo, 5-1-5 Kashiwanoha, Kashiwa, Chiba 277-8564, Japan

**Keywords:** Copepoda, Cyclopoida, Enterognathidae, Seto Inland Sea, symbiosis

## Abstract

A new species of the endoparasitic copepod *Enterognathus* (Cyclopoida, Enterognathidae) is described from a crinoid host in the Seto Inland Sea, western Japan. This is a third species of the genus and its first occurrence in the Pacific Ocean. The new species is distinguished from two previously known congeners by the morphology of the body somites, caudal rami, antennae and legs. Crinoid parasites belonging to *Enterognathus* and the closely related genus *Parenterognathus* have a broad distribution from the northeastern Atlantic through the Red Sea to the West Pacific.

## Introduction

The cyclopoid copepod family Enterognathidae is a compact group accommodating only four genera and six species (Boxshall and Halsey 2004, Ohtsuka et al. 2010). All members are symbionts on Deuterostomia including crinoids, holothuroids and hemichordates (Boxshall and Halsey 2004). Two genera, *Enterognathus* Giesbrecht, 1900 and *Parenterognathus* Ohtsuka, Kitazawa and Boxshall, 2010 are endoparasites of crinoids, and have a wide distribution in the northeastern Atlantic and the Indo-West Pacific regions (Ohtsuka et al. 2010).

During a research cruise in the Seto Inland Sea, western Japan in 2011, we found an undescribed species of the genus *Enterognathus* in a benthic sample. The genus has hitherto consisted of only two species, *Enterognathus comatulae* Giesbrecht, 1900 from the northeastern Atlantic and *Enterognathus lateripes* Stock, 1966 from the Red Sea (Giesbrecht 1900, Stock 1966, Illg and Dudley 1980, Boxshall and Halsey 2004). This is its first occurrence in the Pacific Ocean. The present paper provides a detailed description of the new species with some zoogeographical notes on the genus.

### Material and methods

A juvenile of the crinoid genus *Lamprometra* sp. (cf. Clark 1941, Messing 1997) was collected by a sledge net (mesh size 0.3 mm) at depths of 46.7–46.9 m in the central part of the Seto Inland Sea, western Japan (34°0.590'N, 132°44.32'E–34°0.599'N, 132°44.35'E) on November 7, 2011 (local time 1153–1158). A single specimen of the originally endoparasitic copepod described herein was attached to the outside of the host crinoid, possibly due to an accidental ejection from the intestine of the host during collection. It was preserved in 70% ethanol immediately after capture.

Terminology follows Huys and Boxshall (1991). The type specimen is deposited at the Kitakyushu Museum of Natural History and Human History (KMNH IvR). The host crinoid is deposited at the Atmosphere and Ocean Research Institute of the University of Tokyo.

## Taxonomy

### Enterognathus inabai sp. n.

urn:lsid:zoobank.org:act:E102BE09-F9D6-42CF-8C1F-5AA04090276D

http://species-id.net/wiki/Enterognathus_inabai

Figs 1 –2

#### Material examined.

 An adult female found from the crinoid *Lamprometra* sp. collected from the central part of the Seto Inland Sea, western Japan (34°0.590'N, 132°44.32'E–34°0.599'N, 132°44.35'E), at depths of 46.7–46.9 m, November 7, 2011.

#### Holotype.

 ♀, partly dissected, with appendages on 5 slides and body in a vial (KMNH IvR 500,539).

#### Description.

**Figure 1.**
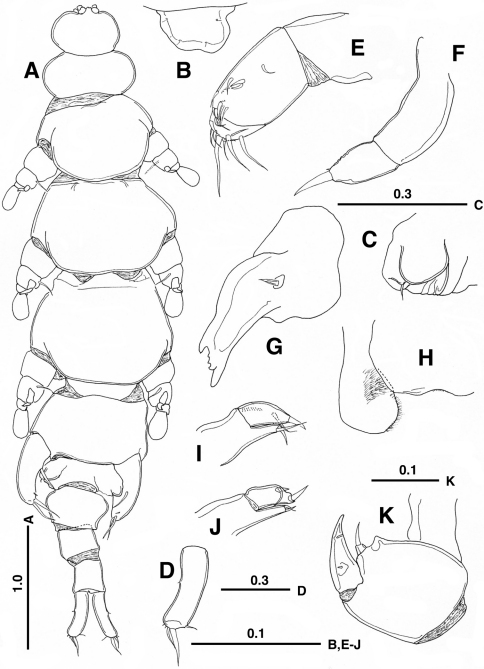
*Enterognathus inabai* sp. n. holotype female: **A** Habitus, dorsal view **B** Rostrum, dorsal view **C** Genital opening, right, dorsal view **D** Caudal ramus, left, dorsal view **E** Antennule **F** Antenna **G** Mandible **H** Labrum and paragnath, ventral view **I** Maxillule **J** Other maxillule **K** Maxilla. Scales in mm.

Female. *Body* (Fig. 1A) 5.17 mm long, from anterior tip of rostrum to caudal ramus excluding caudal setae, flattened dorso-ventrally, weakly sclerotized, elongate, but tagmosis clearly defined. Cephalosome ca. 1.2 times wider than long; rostrum (Fig. 1B) defined basally, slightly asymmetrical with 2 pairs of hair-sensilla. First to fifth pedigerous somites about 2.5, 1.6, 1.6, 1.2 and 1.7 times wider than long, respectively; fourth pedigerous somite (slightly twisted toward right side in Fig. 1A) exhibiting maximum width; genital double-somite protruded laterally into triangular process; each genital opening (Fig. 1C) covered with operculum representing leg 6 and armed with minute seta; single copulatory pore possibly located on posteroventral surface as in *Enterognathus comatulae* (see Fig. 4 in Giesbrecht 1900) and *Parenterognathus troglodytes* (see Fig. 2M in Ohtsuka et al. 2010), but not clearly seen due to damage. First post-genital somite expanded anterolaterally; second and third (anal) free abdominal somites nearly as long as wide. Caudal rami (Fig. 1A, D) symmetrical, slightly curved outward, about 3.7 times as long as wide; caudal setae I to III rudimentary, IV slender, V thick and VI positioned subterminally.

*Antennule* (Fig. 1E) short, 3-segmented; first segment unarmed; second segment longest, with 10 short setae; third segment with 11 setae. *Antenna* (Fig. 1F) short, 2-segmented; basal segment long, unarmed; distal segment short, with 1 short seta and 1 rudimentary seta at tip. *Mandible* (Fig. 1G) with heavily sclerotized gnathobase; cutting edge with large and dorsal and ventral teeth and 2 smaller teeth; palp represented by simple seta. *Labrum* (Fig. 1H) with concave posterior margin. *Paragnath* (Fig. 1H) large, expanded distally, hirsute along inner margin. *Maxillule* (Fig. 1I) 2-segmented; proximal segment bearing praecoxal endite armed with 1 spiniform element and short seta distally; distal segment with 1 subterminal seta, 1 distal spine and row of spinules; other member of pair (Fig. 1J) abnormal, bilobed, with 2 spiniform elements and seta. *Maxillae* (Fig. 1K) connected by intercoxal sclerite; syncoxa with triangular process and single endite furnished with distal seta; basis with stout spine terminally; endopod represented by rudimentary seta. *Maxilliped* absent.

**Figure 2.**
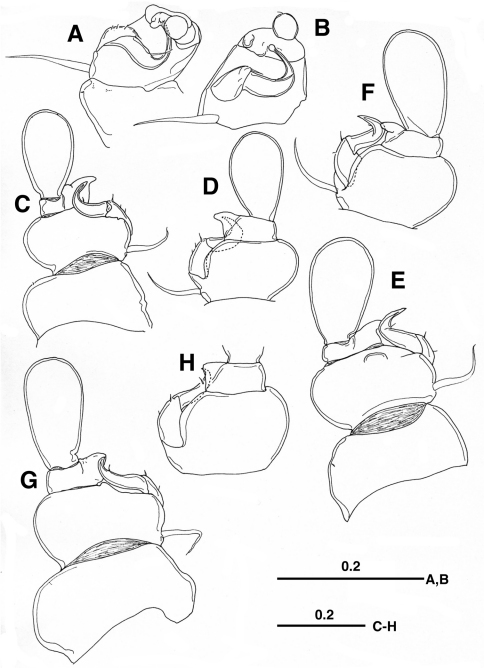
*Enterognathus inabai* sp. n. holotype female: **A** Leg 1, posterior view **B** Leg 1 excluding coxa (more or less flattened), posterior view **C** Leg 2, posterior view **D** Leg 2 excluding coxa, anterior view **E** Leg 3, posterior view **F** Leg 3 excluding coxa, anterior view **G** Leg 4, posterior view **H** Leg 4 excluding coxa and second endopodal segment, anterior view. Scales in mm.

*Legs 1–4* biramous, with 2-segmented rami. Legs 1 and 2–4 ventrally and ventrolaterally positioned on pediger, respectively. Leg 1 (Fig. 2A, B) with minute outer setule on coxa; basis furnished with developed naked outer seta basally; first exopodal segment with 3 setules and longer distal setal element on outer margin, second segment sickle-shaped, abruptly curved outward, terminated at round tip, with naked seta at outer midlength; first endopodal segment broad, with 2 rounded projections, second segment bulbous, with minute seta terminally. *Legs 2–4* (Fig. 2C–H) similar to each other, but gradually increasing in size; first exopodal segment bearing 3 or 2 setules/setal elements in legs 2 and 3–4, respectively; second exopodal segment curved outward, sharply pointed, with minute setule midway or subterminally; first endopodal segment produced outward into triangular process; second endopodal segment spatulate, unarmed.

*Leg 5* (Fig. 1A) 1-segmented, with 1 basal and 2 terminal, developed setae and 1 minute seta subterminally. *Leg 6* (Fig. 1C) represented by genital operculum bearing minute seta.

*Male* unknown.

#### Etymology.

The new specific name “*inabai*” is named in honor of the late emeritus Professor Akihiko Inaba (Hiroshima University) who made great contributions to the faunistic surveys of the Seto Inland Sea (Inaba 1983, 1988).

#### Comparison.

 The present new species is more closely related to *Enterognathus lateripes* from the Red Sea than to *Enterognathus comatulae* from the northeastern Atlantic in sharing synapomorphies such as reductions in segmentation and setation: (1) only one developed seta on the caudal ramus (2 developed setae in *Enterognathus comatulae*); (2) 2-segmented antenna lacking a basal seta (3-segmented, with a single seta on the first segment); (3) a single element on the maxillary basis (2 elements); (4) fewer elements on the distal endopodal and exopodal segments of leg 1 (more elements); (5) 3 developed setae on the fifth leg (4 developed setae).

However it is readily distinguished from *Enterognathus lateripes* in the following features: (1) pedigers 2–5 wider than long (longer than wide in the latter); (2) the first post-genital somite much wider than long (about as long as wide); (3) the second and third post-genital somites about as long as wide (longer than wide); (4) the caudal ramus with 6 setae (4 setae); (5) the terminal seta of the antenna shorter than the second segment (longer); (6) the fifth leg armed with 3 developed setae and 1 minute setule (3 developed setae only); (7) the shape of the distal endopodal segments of legs 1 and 2–4 bulbous and spatulate, respectively (more or less irregular-shaped).

Members of the Enterognathidae have been characterized by the possession of a maximum of 4 setae on the female caudal ramus (see Boxshall and Halsey 2004). However the new species clearly bears 6 setae on each caudal ramus. It is probable that highly reduced setae such seta I have been overlooked in previous works. In addition, the endopods of legs 1–4 of *Enterognathus lateripes* seem to have been misinterpreted by Stock (1966). Stock (1966) interpreted a projection between both rami as originating from the basis, but it is revealed in the present study that it comes from the first endopodal segment.

Giesbrecht’s (1900) descriptions of adults and copepodid stages of *Enterognathus comatulae* are so elaborate that we can provide some morphological and evolutionary comments on the genus. A copulatory pore is located ventrally in the middle of the genital double-somite in this species (Fig. 4 in Giesbrecht 1900). Although the area around the copulatory pore of the new species is damaged, it seems to be located as in *Enterognathus comatulae*, in consideration of the configuration of the paired gonopores. As already pointed out by Stock (1966), 4 post-genital somites are clearly illustrated in *Enterognathus comatulae* (Ab2-5 in Fig. 1 in Giesbrecht 1900). Giesbrecht (1900) seems to have misinterpreted his “second abdominal” somite (Ab2) as a real somite, possibly due to a clear suture line separating it from the genital somite. If a separate genital somite is retained, this might indicate that a reversal or secondary separation of the first post-genital somite from the genital somite has occurred as known in some other copepods (Huys and Boxshall 1991). The caudal ramus of the third copepodid stage of *Enterognathus comatulae* (Fig. 3 in Giesbrecht 1900) is similar to that of *Enterognathus inabai*, suggesting that this character might be neotenic.

#### Zoogeography.

Ohtsuka et al. (2010) pointed out that the genera *Enterognathus* and *Parenterognathus* whose hosts are shallow- to deep-water crinoids are distributed in the northeastern Atlantic through the Red Sea to the West Pacific, and that their origin could have dated back to the early late Paleocene. The present discovery shows that the genus *Enterognathus* alone shows this broad distributional pattern in the Indo-West Pacific plus the northeastern Atlantic. These endoparasitic genera seem to have originated from warm, shallow waters along the southern Eurasian coast of the Tethys Sea in the Paleocene when the North American continent was already separated and located far from Eurasian continent, and simultaneously expanded their horizontal distribution to both the northeastern Atlantic and the Indo-West Pacific since then, as inferred by Ho (1988) for the commensal harpacticoid *Sunaristes* Hesse, 1867 which exhibits a similar modern distribution pattern. In addition, it may be that the ancestor of enterognathids had vertically colonized new frontiers or deep waters since then, because the monotypic *Parenterognathus* was collected from depths of 775–787 m (cf. Ohtsuka et al. 2010). Various symbioses between copepods and invertebrate hosts could have been newly established in shallow- to deep-waters since the Paleocene.

##### Key to species of Enterognathus (females only)

**Table:** 

1	Two well developed setae on the caudal ramus; antenna 3-segmented with a single seta on first segment; 4 developed setae on fifth leg; body length <5 mm (3.8–4.5mm)	*Enterognathus comatulae* Giesbrecht, 1900
–	Only a single well developed seta on the caudal ramus; antenna 2-segmented without a basal seta; 3 developed setae on fifth leg; body length >5 mm	2
2	Pedigers 2–5 wider than long; first post-genital somite much wider than long; second and third free abdominal somites about as long as wide; fifth leg armed with 3 developed setae and 1 minute setule; body length ca. 5.2 mm	*Enterognathus inabai* sp. n.
–	Pedigers 2–5 longer than wide; first post-genital somite as long as wide; second and third free abdominal somites longer than wide; fifth leg furnished with 3 developed setae only; body length ca. 6.1–6.3 mm	*Enterognathus lateripes* Stock, 1966

## Supplementary Material

XML Treatment for Enterognathus inabai
